# Couple Therapy With MDMA—Proposed Pathways of Action

**DOI:** 10.3389/fpsyg.2021.733456

**Published:** 2021-11-11

**Authors:** Anne C. Wagner

**Affiliations:** Remedy, Toronto, ON, Canada

**Keywords:** MDMA, couple, therapy, relationship satisfaction, psychedelic

## Abstract

MDMA's first identified potential as a therapeutic catalyst was for couple therapy. Early work in the 1970s and 1980s explored its potential amongst seasoned psychotherapists and their clients. With the completion of the first pilot trial of MDMA-assisted psychotherapy with couples for PTSD, and as the possibility of conducting MDMA-assisted psychotherapy trials expands due to new regulatory frameworks, we have an opportunity to explore and investigate how and why MDMA-assisted couples therapy works. This theoretical paper will explore the neurobiological and neurochemical effects of MDMA in a relational context, the emotional, behavioral, cognitive and somatic effects within a dyadic frame, and how empathy, communication, perception of social connection/support, non-avoidance, openness, attachment/safety, bonding/social intimacy and relationship satisfaction, are all impacted by MDMA, and can be harnessed to facilitate systems-level and interpersonal healing and growth. A model to support MDMA-assisted couple therapy is introduced, and future directions, including implications for intervention development and delivery, will be elucidated.

## Introduction

Couple therapy offers the opportunity to heal, grow and change while in relationship. Given that people do not exist in isolation, it creates the context to change in the milieu in which people live their lives. The current paper offers an examination of the reasons for why, and how, a transtheoretical model of MDMA-assisted couple therapy may be an important contribution to couple therapy treatment options. This mini review provides an overview of MDMA, how MDMA functions in an interpersonal context, including its neurochemical, psychological and subjective effects, and how these effects translate into therapeutic outcomes in a transtheoretical approach to MDMA-assisted couple therapy. Potential pathways of action are introduced, and a model to support the enhancement of these treatment outcomes is outlined.

Couple therapy can be utilized for primarily relationally-oriented presenting problems (e.g., relationship distress, difficulties with communication, relationship enhancement, coping with infidelity, abusive interactions, transgressions of trust, life transitions, intimacy, etc.), for relationship enhancement (e.g., to prepare for a life change, such as the birth of a child or marriage), and for focusing on mental health difficulties in one or both partners (e.g., couple treatment for PTSD, depression, OCD, etc.). Findings from dyadic interventions for specific diagnoses demonstrate improvement in both mental health outcomes (OCD, PTSD, depression), relationship satisfaction and functioning for both partners (Baucom et al., 1998, 2012; Christensen and Heavey, 1999; Benson et al., 2012; Monson et al., 2012; Fischer et al., 2016). Each of these presenting foci can be approached from a variety of therapeutic modalities (e.g., cognitive-behavioral, integrative, emotion-focused, systems-focused, psychodynamic) (e.g., Johnson and Greenberg, 1988; Baucom et al., 1998; Christensen et al., 2020). Common factors among effective couple therapies include objective, shared understanding of the presenting concerns, decreasing emotion-driven dysfunctional behavior, increasing emotion-driven avoided behavior, improving communication and bolstering strengths and gains (Benson et al., 2012). The impact of relational distress has ramifications not only on the relationship, but also each partner's mental health and the functioning of broader family systems (Atkins et al., 2009; Baucom et al., 2012). While couple therapy can reduce relationship distress and increase relationship satisfaction, many couples do not benefit or may not maintain gains over time (Baucom et al., 1998; Christensen et al., 2010). Alternative couple treatment options which can enhance common effective factors are therefore needed.

## MDMA

MDMA (3,4-methylenedioxymethamphetamine) is a ring-substituted phenylisoproylamine derivative that increases the release and prevents reuptake of serotonin, norepinephrine, and dopamine (Liechti and Vollenweider, 2001; Farré et al., 2004; Hysek et al., 2011; Bershad et al., 2016). The 5-HT2 receptor plays a contributing role in its effect (Liechti et al., 2000; van Wel et al., 2011). MDMA elevates serum oxytocin (Wolff et al., 2006; Dumont et al., 2009) and vasopressin (Bershad et al., 2016), as well as cortisol and prolactin (Grob et al., 1996; Mas et al., 1999; Harris et al., 2002). MDMA causes a decrease in cerebral blood flow to the amygdala and hippocampus, an increase in activity in the prefrontal cortex, action in the occipital cortex and insula, and a decrease in functional connectivity between the hippocampus and prefrontal cortex, and an increase between the hippocampus and amygdala (Gamma et al., 2000; Phelps et al., 2001; Carhart-Harris et al., 2014, 2015; Walpola et al., 2017; Feduccia and Mithoefer, 2018).

## MDMA-Assisted Couple Therapy

MDMA-assisted couple therapy was conducted prior to MDMA being made illegal in the mid-1980s. Greer and Tolbert (1986, 1998) conducted a series of cases of MDMA-assisted psychotherapy, including with couples, that demonstrated improvements in fear of emotional hurt and improved communication and introspection. Reports by Sasha and Ann Shulgin describe MDMA's therapeutic use with couples shortly after it was re-synthesized in the mid-1970s (e.g., Holland, 2001), and that it is an excellent tool for communication and to navigate relational issues. Reports suggest that MDMA had broad use by psychotherapists in non-research settings (e.g., Passie, 2018). With recent re-initiation of MDMA-assisted therapy research, long term follow-ups of individual MDMA-assisted psychotherapy for posttraumatic stress disorder (PTSD) demonstrate that almost two thirds of participants reported improved relationships with loved ones following treatment (Jerome et al., 2020). One pilot trial has been conducted for MDMA-assisted Cognitive Behavioral Conjoint Therapy (CBCT) for PTSD, wherein both partners participated in the entire protocol, including MDMA-assisted sessions. Findings from the study include improved PTSD scores, relationship satisfaction, posttraumatic growth, and improved social intimacy for the partner with PTSD (Monson et al., 2020; Wagner et al., 2021). These pilot trial results are promising, and suggest the potential for non-diagnostic specific MDMA-assisted couple therapy, given the significant improvements in relationally oriented outcome measures.

## MDMA'S Pharmacological Effects and Couples

MDMA's empathogenic qualities have made it a prime candidate as an adjunct to psychotherapy. When considering the couple therapy context, understanding the neurochemical experience related to romantic love illuminates this potentially catalytic combination. The hormones and neurotransmitters most closely associated with the experience of love include oxytocin and vasopressin (linking to attachment and bonding), dopamine and serotonin (causing pleasure and positive mood), and the brain areas most impacted include the amygdala (registering threat, happiness and fear), prefrontal cortex (related to reasoning) and the hippocampus (engagement with thoughts and memories), as well as the caudate nucleus (registering love) and the hypothalamus (registering lust) (e.g., Zeki, 2007; Fisher et al., 2010).

The similarities in these areas of action, and notably the release of oxytocin (which can help facilitate interpersonal trust, attachment, bonding, forming affection) (Kosfeld et al., 2005; Bartz and Hollander, 2006; Domes et al., 2007), notably with its interaction with vasopressin and its context-dependent nature (Carter et al., 2020), and serotonin (increasing positive mood and extraversion, reducing depression and anxiety) (e.g., Hasler et al., 2009; van Wel et al., 2012), demonstrate that similar processes are activated in the neurobiology of love as are with MDMA. Notably, several studies have failed to show a relationship between the social effect of MDMA and oxytocin (e.g., Kuypers et al., 2017), which suggests that the empathogenic effects of MDMA are not unifactorial to the presence of oxytocin. Using the context of an MDMA-assisted psychotherapy session to work with a romantic relationship can therefore allow a potential reactivation or “remembering” of these neurochemical pathways to occur. Additionally, the increase in cortisol and noradrenergic response with MDMA can raise levels of arousal (Mas et al., 1999; Harris et al., 2002; Hysek et al., 2011, 2012a), which may support motivation to engage in therapy and interactions with the partner.

## Pathways of Action for MDMA-Assisted Couple Therapy

Synthesizing the literature on the psychological, subjective and perceived effects of MDMA, the following offers a distillation of pathways in which MDMA may assist couple therapy. Four broad areas of psychological impact (emotion, cognition, behavior, somatic experience) highlight eight cross-therapeutic outcomes for MDMA-assisted couple therapy: (1) empathy, (2) communication, (3) perception of social connection/support, (4) non-avoidance, (5) openness, (6) attachment/safety, (7) bonding/social intimacy, (8) relationship satisfaction (see Table 1 for synthesis).

**Table 1:** Areas of focus for MDMA-assisted couple therapy outcomes.

**Area of focus**	**How MDMA supports**	**Proposed therapeutic interventions to facilitate**
Empathy	Oxytocin release helps increase interpersonal focus, feelings of interpersonal trust, social affiliation (Kosfeld et al., 2005; Bartz and Hollander, 2006; Domes et al., 2007). MDMA associated with seeing others as empathetic and caring (Hysek et al., 2013; Bedi et al., 2014; Wardle and de Wit, 2014), and increases emotional empathy beyond oxytocin alone (Kuypers et al., 2014)	Centering both peoples' experiences, sharing of feelings
Communication	MDMA associated with greater interpersonal focus in language (Bedi et al., 2014), reduction in reactivity to angry facial expressions and greater reward in happy faces (Bedi et al., 2009)	Present and practice skills of sharing and listening with both negative and positive content
Perception of social connection and support	Reduction in feeling of social pain (Frye et al., 2014), decreased feelings of threat, increased feelings of interpersonal trust (Kosfeld et al., 2005; Domes et al., 2007), increased identification of prosocial feelings (Bedi et al., 2010)	Highlight strength in couples' interactions, remind in integration to reduce likelihood of returning to old patterns after MDMA sessions
Non-avoidance	MDMA assists in fear attenuation, allowing for approach of difficult experiences and memories (Young et al., 2015, 2017; Doss et al., 2018; Feduccia and Mithoefer, 2018; Hake et al., 2019)	Explain why non-avoidance is helpful-while difficult initially, is supportive of growth of relationship to not allow difficulties to expand or root over time
Openness	MDMA can assist in increased openness to experience and decreased neuroticism (Wagner et al., 2017). The ability to engage in interactions may be supported by the release of cortisol, and paired with oxytocin (e.g., Mas et al., 1999; Hysek et al., 2011)	Creating shared intentions, and establishing as a value to bring through the process
Attachment/ safety	Decreased feelings of threat, increased feelings of interpersonal trust (Kosfeld et al., 2005; Domes et al., 2007)	Skills to engage in difficult conversations, take breaks and re-engage. Creating a template for future experiences
Bonding/ social intimacy	Oxytocin, which MDMA causes to release, supports feelings of social bonding (Bartz and Hollander, 2006). MDMA helps increase cooperation and feelings of trustworthiness (Stewart et al., 2014; Gabay et al., 2019). Increased experience of social intimacy following MDMA-assisted couple therapy (Wagner et al., 2021)	Couple engaging in the whole process together
Relationship satisfaction	Improved relationship satisfaction, decreased distress in MDMA-assisted couple therapy (Monson et al., 2020; Wagner et al., 2021)	Encouraging shared experiences, engagement together in the therapeutic process

### Emotions

In experimental contexts of emotion processing, including fMRI, psychophysiological measures and self-report studies, MDMA has been found to facilitate the perception of positive emotional expressions, empathy, and reduce the social pain associated with negative emotional expressions perceived from others and social rejection (Bedi et al., 2009; Hysek et al., 2012b, 2014; Frye et al., 2014). MDMA has been found to improve identification of emotions in others, as well as increase prosocial feelings (Bedi et al., 2010). MDMA has also been found to increase attention to positive emotional cues (Bershad et al., 2019). In therapeutic contexts, MDMA allows for the experiencing of challenging emotions, as well as increased compassion and decreased defensiveness (e.g., Metzner and Adamson, 2001; Stolaroff, 2004; Feduccia and Mithoefer, 2018; Wagner et al., 2019). MDMA has also demonstrated a reduction in anxiety in therapeutic contexts (e.g., Danforth et al., 2018). Evidence demonstrates that MDMA enhances feelings of closeness to others (Borissova et al., 2020), which is particularly useful in couple therapy to allow the individuals to feel connected within the experience, regardless of content being shared. In couple therapy, facilitating the sharing of emotional content leads to improved closeness and satisfaction (e.g., Sanford, 2007; Christensen et al., 2020). MDMA can support this experience. Painful feelings can be seen as useful in the therapeutic process, feelings of love and deep appreciation can emerge, and attenuating the fear response to threat of emotional integrity can help support the full experience of emotion by reducing defensiveness (Metzner and Adamson, 2001; Stolaroff, 2004). MDMA is associated with seeing others as empathetic and caring, beyond the effects of oxytocin release alone (Hysek et al., 2013; Bedi et al., 2014; Kuypers et al., 2014; Wardle and de Wit, 2014). The experience of feeling empathetic toward oneself and others when in an MDMA session allows for an unfettered experience of the emotion and present moment, which outside of this context often gets marred by the presence of old memories, the drive to be right or heard, or distress, pulling the person out of the moment.

### Cognitions

The perceived psychological cognitive effects of MDMA include being able to engage with difficult memories, likely due to a reduction in perceived threat and increased interpersonal trust (Kosfeld et al., 2005; Domes et al., 2007), as well as having clarity of thought without becoming overwhelmed by the emotions that the thoughts may typically elicit (Wagner et al., 2019). Neuroimaging results demonstrate that negative memories may be perceived as more tolerable following MDMA (Carhart-Harris et al., 2014). Additionally, memories, including those with strong emotional content, can be engaged with differently and fear extinction can occur when engaged in the memory (Young et al., 2015, 2017; Doss et al., 2018; Feduccia and Mithoefer, 2018; Hake et al., 2019). MDMA also increases reported perceptions of trust (Stewart et al., 2014), a salient cognitive process in interpersonal dynamics that can perpetuate significant relational distress. There is evidence that there is a reopening of a critical learning period for social interaction with MDMA in mice, allowing for new learning about social interactions to occur (Nardou et al., 2019). If translatable to humans, this offers an immense opportunity for new learning to occur, which then spans into the next category, behavior.

### Behaviors

MDMA has been shown to alter speech, increasing social and sexual words and a willingness to disclose personal information (Baggott et al., 2015), and an increase in interpersonal focus in language (Bedi et al., 2014). MDMA has also been shown to increase cooperation (Gabay et al., 2019). Therapeutically, MDMA can create a reduction in experiential avoidance and engagement in challenging content (emotional and cognitive) without disorientation, including a desire to communicate (Feduccia and Mithoefer, 2018; Wagner et al., 2019). Increased openness to experience, which can facilitate greater engagement and risk-taking in vulnerability in interpersonal relationships, has been shown to increase following MDMA-assisted psychotherapy (Wagner et al., 2017). Reports of increased introspection and improved communication collectively create a behaviorally rich opportunity for relational processing and engagement for the couple (e.g., Metzner and Adamson, 2001; Stolaroff, 2004; Wagner et al., 2019).

### Somatic Experiences

The somatic effects of MDMA, specifically linked to psychological processes, can include strong physiological sensations related to past experiences, which are helpful to re-experience in the supportive container of the therapeutic setting (Mithoefer et al., 2014). Somatic effects can be linked to any of the cognitive or emotional content arising, including memories (Wagner et al., 2019). Additionally, soothing or calming somatic effects can occur, which can offer supportive experiences for the couple. Reduced fear response and increased feelings of closeness may help amplify these supportive somatic experiences, and allow for immersion into difficult ones as needed. Results from brain imaging after MDMA demonstrate greater reward from happy facial expressions and less reactivity to angry facial expressions (Bedi et al., 2009), potentially supporting the tolerance of emerging somatic experiences.

## A Proposed Transtheoretical Model For MDMA-Assisted Couple Therapy—The Set Model

The SET Model (Setting, Structure and Safety, Exploration and Experience, Template and Trust) is designed to capture the essential components of MDMA-assisted couple therapy, regardless of theoretical approach and across a range of treatment targets. Principles are derived from effective couple therapy (Benson et al., 2012) and the unique MDMA experience, both from the literature and from the author's experience as an investigator of MDMA-assisted CBCT.

### Structure of Therapy

**Figure 1:**
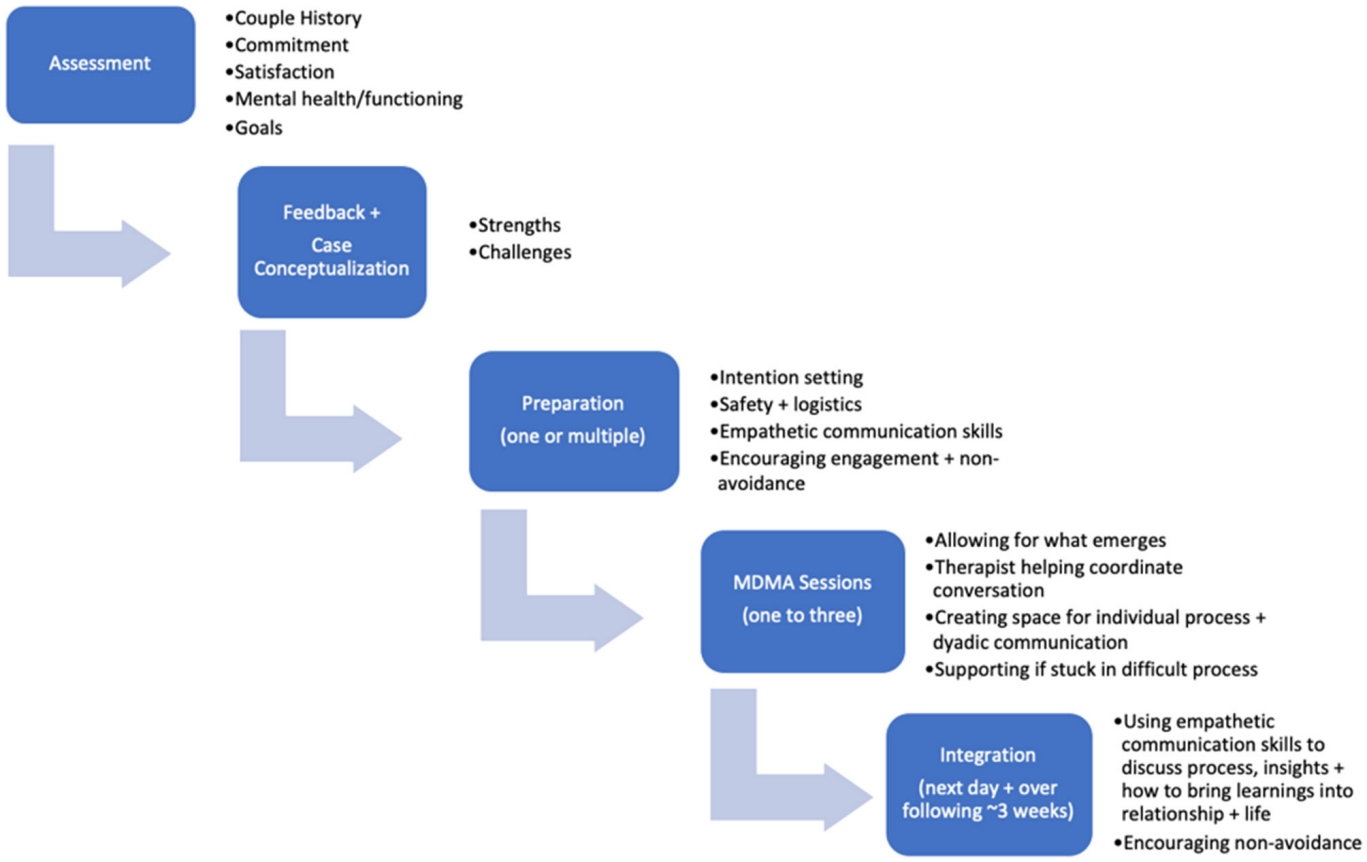
MDMA-assisted couple therapy structure.

Functionally, it is recommended that practitioners begin with a thorough assessment process to understand the commitment levels and distress of the clients, any co-occurring mental health challenges, the couples' history and to understand their goals for the therapeutic process. Following assessment, feedback and a collaborative case conceptualization with the couple are recommended to understand their particular strengths and challenges. Preparation for the MDMA-assisted sessions would then occur across a series of psychotherapy sessions (ranging from one multi-hour session to several shorter sessions, depending on the needs of the clients). Preparation consists of creating a shared language for the sessions through the learning and practice of empathic communication skills, emphasizing the softening of language to facilitate empathy. Intention setting, and practical preparation for the session should be covered. The MDMA sessions themselves allow for an open experience without agenda, with the therapist(s) serving as facilitator for conversations, and navigating the needs of both individuals to ensure both have the opportunity for deep internal reflection as well as communication together. Integrative sessions allow for processing of the experience in the MDMA sessions, inquiry regarding insights, empathy and shared experiences, and application and exploration of learnings to the couples' stated goals and future. The therapeutic process with MDMA-assisted therapy offers the unique opportunity to create a framework to support an experience that will then be able to serve as a template for future interactions, with MDMA sessions themselves serving as a catalyst. See Figure 1 for a summary of the therapeutic process.

### Principles of Practice

#### Setting, Structure and Safety

Creating a safe setting (physically and emotionally), as has been well described in psychedelic-assisted therapy literature (e.g., Metzner and Adamson, 2001; Mithoefer et al., 2014), is essential for both people to feel comfortable engaging in deep emotional territory and navigating a non-ordinary state. For couples, this includes discussions of safety related to disagreements (encouraging a process of stopping disagreements before they escalate and returning to conversations when they are more settled), and of no interpersonal violence. This also extends to safety and the structure of approaching the experience together, including the development of shared intentions for the sessions, and the safety of knowing they will have space to integrate together afterwards. Introducing and practicing communication skills can allow for a shared language and understanding of how to communicate effectively. This structure may allow for a deeper experience in the MDMA session, knowing there is a scaffold to tether to in terms of how to discuss what emerges, which can help mitigate fear, and create safety. Creating the structure that both peoples' experiences are central (that there is no “patient” and that both people are equally participant in the experience) can help mitigate power dynamics and create more opportunity for both people to engage fully.

#### Exploration and Experience

Fostering an environment of curiosity, openness and non-avoidance (experientially, emotionally and behaviorally to support the relationship and prevent difficulties rooting over time) in preparation before, during the MDMA session, and throughout integration, encourages a full experience of emotions, their expression and communication. Facilitating the process of recapping the insights, shifts, and gains throughout the therapeutic process can help orient the couple to their unfolding experience, and can help solidify their learnings, as there can be a tendency to revert to old patterns in any couple process over time. Encouraging the exploration of difficult conversations and highlighting the shared experience they are having can help to create the conditions for empathy and bonding, as well as perceptions of social support.

#### Template and Trust

Through this process, the couple is creating a template for how to communicate effectively, how to debrief together, and how to navigate intense emotional experiences for the future. By establishing a stance of fostering trust and openness to whatever emerges in the therapy process, this can help model the experience for their relationship. Supporting non-avoidance and highlighting strengths in the relationship, as well as their ability to navigate stressors, can help foster trust in themselves and each other.

## Discussion

This synthesis of the literature demonstrates the potential usefulness of MDMA as a catalyst to couple therapy, given its neurochemical actions, and emotional, cognitive, behavioral and somatic effects. While preliminary research has been conducted using one model of therapy (CBCT for PTSD) with MDMA (Monson et al., 2020), the SET model proposes a larger, overarching set of principles that can be applied irrespective of therapeutic modality and for different therapeutic targets beyond PTSD. These principles are suggested as common factors that can enhance and guide the MDMA-assisted couple therapy experience, and future research is needed to test this framework in application.

Additionally, future research can investigate, in both interventional and experimental contexts, the neurochemical and subjective experiences of MDMA in a dyadic context, as the majority of research has been conducted with individuals, not as couples. This synthesis also has implications for treatment development, including the testing of MDMA-assisted couple therapy for the reduction of relationship distress and enhancement of relationship satisfaction. In the recent resurgence of MDMA-assisted therapy research, the majority has been to treat psychiatric disorders, such as PTSD, social anxiety and anxiety related to life-threatening illness (Danforth et al., 2018; Mithoefer et al., 2019; Wolfson et al., 2020). MDMA-assisted couple therapy is a prime opportunity to extend these investigations beyond psychiatric diagnosis, while targeting outcomes that have broad and important impact on peoples' lives.

Notably, very limited MDMA-assisted couple therapy has been conducted in a research context, and therefore this mini review and proposed model is limited in scope to theorized mechanisms and pathways of action, and is preliminary in nature.

Altogether, this review suggests that the neurobiological and neurochemical effects of MDMA in a relational context, and the emotional, behavioral, cognitive and somatic effects within a dyadic frame guided by the SET model can be harnessed to facilitate meaningful interpersonal healing and growth.

## Author Contributions

The author confirms being the sole contributor of this work and has approved it for publication.

## Conflict of Interest

The author declares that the research was conducted in the absence of any commercial or financial relationships that could be construed as a potential conflict of interest.

## Publisher's Note

All claims expressed in this article are solely those of the authors and do not necessarily represent those of their affiliated organizations, or those of the publisher, the editors and the reviewers. Any product that may be evaluated in this article, or claim that may be made by its manufacturer, is not guaranteed or endorsed by the publisher.
